# Recombinant Viruses Initiated the Early HIV-1 Epidemic in Burkina Faso

**DOI:** 10.1371/journal.pone.0092423

**Published:** 2014-03-19

**Authors:** Peter N. Fonjungo, Marcia L. Kalish, Amanda Schaefer, Mark Rayfield, Jennifer Mika, Laura E. Rose, Orville Heslop, Robert Soudré, Danuta Pieniazek

**Affiliations:** 1 HIV and Retrovirology Branch, Division of AIDS, STD, and TB Laboratory Research, National Center for Infectious Diseases, Atlanta, Georgia, United States of America; 2 Division of Global HIV/AIDS, Center for Global Health, Centers for Disease Control and Prevention, Atlanta, Georgia, United States of America; 3 Unité de Formation et de Recherche en Sciences de la Santé (UFR/SDS), Université de Ouagadougou, Ouagadougou, Burkina Faso; Chinese Academy of Sciences, Wuhan Institute of Virology, China

## Abstract

We analyzed genetic diversity and phylogenetic relationships among 124 HIV-1 and 19 HIV-2 strains in sera collected in 1986 from patients of the state hospital in Ouagadougou, Burkina Faso. Phylogenetic analysis of the HIV-1 *env* gp41 region of 65 sequences characterized 37 (56.9%) as CRF06_cpx strains, 25 (38.5%) as CRF02_AG, 2 (3.1%) as CRF09_cpx, and 1 (1.5%) as subtype A. Similarly, phylogenetic analysis of the protease (PR) gene region of 73 sequences identified 52 (71.2%) as CRF06_cpx, 15 (20.5%) as CRF02_AG, 5 (6.8%) as subtype A, and 1 (1.4%) was a unique strain that clustered along the B/D lineage but basal to the node connecting the two lineages. HIV-2 PR or integrase (INT) groups A (n = 17 [89.5%]) and B (n = 2 [10.5%]) were found in both monotypic (n = 11) and heterotypic HIV-1/HIV-2 (n = 8) infections, with few HIV-2 group B infections. Based on limited available sampling, evidence suggests two recombinant viruses, CRF06_cpx and CRF02_AG, appear to have driven the beginning of the mid-1980s HIV-1 epidemic in Burkina Faso.

## Introduction

Acquired immunodeficiency syndrome (AIDS) is caused by two genetically distinct types of human immunodeficiency virus (HIV), HIV type 1 (HIV-1) and HIV type 2 (HIV-2). HIV-1 consists of four major groups, M, N, O and P, each resulting from separate cross-species transmissions from chimpanzees or gorillas to humans [1]–[5]. Within the Group M viruses, phylogenetic analysis has identified 12 subtypes and sub-subtypes (A1, A2, A3, B, C, D, F1, F2, G, H, J and K), at least 57 circulating recombinant forms (CRFs), and innumerable unique recombinant forms, which encompass the majority of HIV-1 infections worldwide [6]. The level of infections with group O viruses has remained very low and they are found mostly in West-Central Africa [7], [8]. Currently, very few cases of HIV-1 groups N and P have been identified, all in persons from Cameroon [4], [9]–[14].

Similarly, HIV-2, which is the result of 8 separate cross-species infections from sooty mangabey monkeys to humans, has been classified into genetically distinct groups designated A to H and are found primarily in West African countries, such as Guinea-Bissau, Senegal, Ivory Coast, Sierra Leone, Ghana, Mali, and The Gambia [15]–[18], but has spread to other non-African countries [19], [20]. HIV-2 group A is most prevalent, followed by group B. HIV-2 groups C-H are rare [21], [22].

Although HIV-1 and HIV-2 seroreactivity was first reported in sub-Saharan African countries in the mid-1980s [23]–[25], little genetic characterization has been done on those early HIV strains. Phylogenetic characterization of HIV sequences remains a powerful tool for tracking the evolution and distribution of HIV worldwide. Of critical importance to our understanding the evolution of the HIV pandemic is having access to samples from early in the epidemic, especially from West and Central Africa, which are the epicenters of the HIV-2 and HIV-1 epidemics, respectively [17], [26], [27]. The earliest known HIV-1 sequence (ZR59) came from a 1959 HIV-1 seropositive plasma sample from Zaire, now called the Democratic Republic of Congo (DRC). Phylogenetic analysis of *gag*, *pol*, and *env* genes confirmed it as an HIV-1 group M virus and placed it basal to the node connecting the subtype B and D lineages [28]. The second earliest known HIV sequence was found in a 1960 lymph node biopsy specimen obtained from an adult female in Kinshasa, Zaire (DRC60), and was phylogenetically characterized as HIV-1 group M, subtype A in the *gag*, *pol* and *env* gene regions [29]. Finding viruses from two different HIV-1 subtypes, suggested that significant viral diversification had already taken place by 1960 [29]. Next, 56 samples from Kinshasa, Zaire, collected in the mid-1980s, demonstrated the presence of all the HIV-1 group M subtypes, except B, along with circulating recombinant form (CRF) 01 (CRF01_AE), and many unique recombinant strains [27].

Between 1985-1987 the HIV-2 strains ROD, BEN and ST, isolated from patients with AIDS from Cape Verde, Mali and Senegal, respectively, phylogenetically clustered with HIV-2 group A reference strains [30]-[32], while the HIV-2 strains GH-2, D205 and UC1 (1986–1988) recovered from AIDS patients in Ghana and Ivory Coast, respectively, clustered with HIV-2 group B [32], [33]–[35]. To date, no HIV-2 subtypes have been described for any of the HIV-2 groups.

Early studies on serum specimens from Burkina Faso, collected between 1985–1987, showed an HIV seroprevalence of 1.7%, 1.8%, 4.5% and 14.6% among pregnant women, prisoners, hospital patients and prostitutes, respectively [36]. A study from 1994 showed the HIV prevalence in female sex workers to be as high as 58.2% [37]. The first HIV-1 genetic subtype described from Burkina Faso was in a specimen collected in 1996 [38], [39]. Phylogenetic analysis of the full genome sequence classified the HIV isolate, BFP90, as a complex recombinant, CRF06_cpx involving recombination between at least 4 HIV-1 subtypes: A, G, J and K [38], [39], [40]. Thereafter, additional studies on specimens collected in 1996, 2000, 2001, 2003, and 2004 have confirmed CRF06_cpx to be the predominant strain in Burkina Faso, followed by CRF02_AG, and the less prevalent subtypes A, A3, G, F1, H and CRF09-cpx [41]–[43]. In other West and West-Central African countries, such as in Senegal, Ivory Coast, Nigeria, Gabon, Cameroon, where multiple subtypes and CRFs co-circulate, more recent samplings have reported their predominant HIV-1 strain as CRF02_AG [44]–[46].

In this study we have sequenced and performed phylogenetic analysis on HIV strains from specimens collected in 1986 in the West African city of Ouagadougou, Burkina Faso, in order to characterize the viruses that may have initiated the early epidemic and to understand how the distribution of those strains have evolved.

## Materials and Methods

### Specimens

We received 849 remnant and anonymized serum samples collected in 1986 from patients attending the state hospital in Ouagadougou, the capital of Burkina Faso in West Africa. The amount of the serum samples we received was very small, ranging from 100 μl to about 250 μl. Since both HIV-1 and HIV-2 are found in West Africa, all sera were tested by two separate whole viral lysate EIA assays to detect antibodies to HIV-1 and HIV-2 (Genetic Systems, Redmond, WA, USA). To confirm HIV type, EIA reactive specimens were tested with INNO-LIA HIV-1/2 (INNOGENETICS, Ghent, Belgium), which has synthetic peptides recognizing antibodies to HIV-1 p17, p24, p31, gp41, and gp120 proteins, and to HIV-2 gp36 and gp105 proteins. Western blot was performed on some samples, however, the discrimination between HIV-1 and HIV-2 was based on INNO-LIA HIV-1/2 results as previously described [47], [48].

### Ethical Approval

The initial HIV prevalence survey samples were consented to at the time of blood draw, but there was no formal IRB in Burkina Faso in 1986 and no written informed consent retained. Permission for the survey and specimen collection was granted by the Ministry of Health of Burkina Faso. For purposes of the genetic studies reported here, the archived specimens were received unlinked and anonymized and oversight of their analysis fell under the Global Monitoring for Variants Strains of HIV protocol #1367 approved by the CDC Institutional Review Board.

### RNA Extraction, Amplification and Sequencing

Extraction of RNA from all INNO-LIA positive sera was done using the NucliSens nucleic acid manual or automated protocols (Organon Teknika, Boxtel, the Netherlands). Conditions for RT-PCR and secondary DNA PCR amplifications (RT-PCR/PCR) and the sets of primers for HIV *env* gp41 region, PR, and INT amplifications have been described previously: HIV-1 *env* gp41 (outside: gp40F1/gp41R1 and nested: gp46F2/gp47R2 or gp48R2) [49]; HIV-1 PR (outside: DP10F/DP11R and nested: DP16F/DP17R) [50], [51], HIV-2 PR (outside: DP20F/DP21R and nested: DP26aF/DP27R) [52]; and HIV-2/SIV INT (outside: INT-F1/INT-R1 and nested: INT-F2/INT-R2) [53]. PCR-amplified products were purified with the QIAquick PCR Purification Kit (Qiagen, Valencia, CA USA) and directly sequenced with both forward and reverse nested PCR primers on an automated DNA sequencer (ABI model 377; Applied Biosystems Inc., Foster City, CA).

### Characterization of Specimens

HIV sequences from INNO-LIA positive sera were subjected to RT-PCR/PCR amplification of different regions of the HIV genome. First, HIV-1 seropositive specimens were amplified using *env* gp41 primers that allow amplification of HIV-1 groups M, N, and O and SIVcpz within a 366-bp fragment [49]. Samples were further screened for HIV-1 using PR primers known to amplify the entire 297-bp PR gene of group M subtypes A-K in field samples with a high efficiency [54], [55]. HIV-2 seropositive specimens were amplified with HIV-2 type-specific PR primers that allow identification of at least the two major groups, A and B [52]. The HIV-2 PCR samples from this collection were further screened for HIV-2 with *pol*-INT primers.

Specimens showing INNO-LIA HIV-1/HIV-2 heterotypic seroreactivity were submitted to separate amplifications with HIV-1 and HIV-2 PR primers, which are type specific and permit selective PCR amplification of HIV-1 and HIV-2 strains from mixed HIV-1/HIV-2 infections [56]. These samples were additionally screened for the presence of HIV-1 with gp41 primers and for HIV-2 with *pol*-INT primers [53]. Sequencing of two different gene regions, especially from opposite ends of the genome, might increase the likelihood of detecting potential HIV-1 intersubtype recombinants. Since our serum samples were more than 20 years old and the HIV RNA was likely degraded, we selected both the HIV-1 gp41 and PR gene regions and the HIV-2 PR and INT genes for initial amplification attempts because they are reasonably conserved genes and fairly short gene regions.

### Phylogenetic Analysis

The derived HIV-1 and HIV-2 DNA sequences were aligned using CLUSTAL W1.83 multiple-sequence alignment program included in the GeneStudio package, which provides the interface to phylogenetic programs on a PC workstation [57]. Alignments included known HIV-1 or HIV-2 sequences extracted from the Los Alamos Laboratory HIV Sequence database [58], representing the different genetic subtypes and the CRFs documented in West Africa. Phylogenetic analysis was performed by the neighbor joining method, with the nucleotide distance calculated by Kimura’s two-parameter model included in the PHYLIP package (version 3.5c) [59] with and without bootstrapping. SIVcpz sequences were used as outgroups. It was further verified with Maximum Likelihood method using FastDNAml program (http://www.genestudio.com).

### Genetic Distance Analysis

Subtype and CRF designations were determined from phylogenetic analysis, and sequences were input into the MEGA program, version 2.0 [60] to calculate the means of the pair-wise genetic differences among each subtype and CRF. Values for the genetic differences between sequences were evaluated based on the distance index that was created from the nucleotide alignment [61]

### Sequence Data

The sequences obtained in this study have been deposited in GenBank under the following accession numbers: AY640416-AY640433 for HIV-2 Protease; AY645781-AY645795 for HIV-1 Protease CRF02; AY662958-AY662982 for HIV-1 env gp41 CRF02; AY928421-AY928457 for HIV-1 env gp41 CRF06.

## Results

### Seroreactivity and RT-PCR Amplification

Of the 849 1986 Burkina Faso specimens from hospitalized patients, 172 (20.3%) were confirmed to be HIV-1 and/or HIV-2 seropositive by the INNO-LIA HIV Line assay. From the 172 seropositive samples, 124 (72.1%) were only HIV-1 RT-PCR/PCR amplifiable; 51 (41.1%) were amplified by gp41 primers, 59 (47.6%) by PR primers and 14 (11.3%) were amplified by both gp41 and PR primers (Table 1). Monotypic HIV-2 was amplified from 11 seropositive samples, 10 (90.9%) in the PR region, and 1 (9.1%) additional sample was amplified in the HIV-2 INT region. Both HIV-1 and HIV-2 were amplified from 8 samples: 7 HIV-1 PR genes and 8 HIV-1 gp41 genes were amplified and all 8 HIV-2 strains were amplified by HIV-2 PR primers.

**Table 1:** Summary of distribution of HIV-1 (env gp41, PR) and HIV-2 (PR and INT) gene sequences.

	HIV-1 Only			HIV-2 Only			HIV-1 and HIV-2			
Viruses	*PR	*gp41	Viruses	*PR	*INT	Viruses	*HIV-1 PR	*HIV-1 gp41	*HIV-2 PR	*HIV-2 INT
n = 11	CRF02	Neg	n = 10	A	Neg	n = 1	CRF02	Neg	A	Neg
n = 39	CRF06	Neg	n = 1	Neg	B	n = 2	CRF06	Neg	A	Neg
n = 4	A	Neg				n = 1	A	Neg	B	Neg
n = 1	B/D	Neg				n = 1		CRF06	A	Neg
n = 20	Neg	CRF02				n = 3	CRF06	CRF06	A	Neg
n = 27	Neg	CRF06								
n = 2	Neg	CRF09								
n = 1	Neg	A								
n = 3	CRF02	CRF02								
n = 6	CRF06	CRF06								
n = 2	CRF06	CRF02								

CRF, denotes circulating recombinant forms; * denotes gene regions where RT-PCR amplification was attempted; PR, protease; INT, denotes Integrase; gp41, envelope glycoprotein 41. Neg indicates no amplifiable PCR product.

### Phylogenetic Analysis of Early Sequences

**Figure 1:**
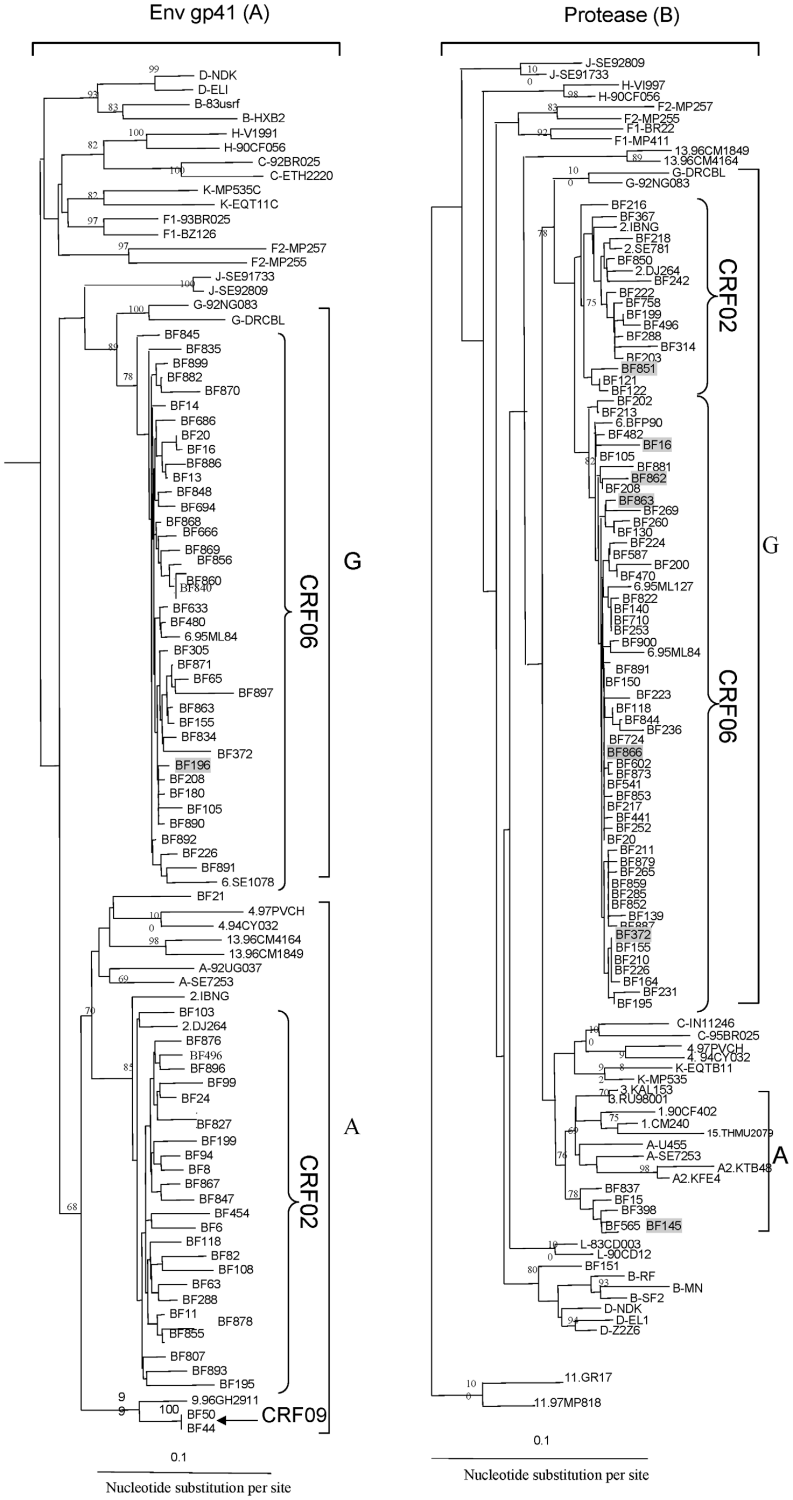
Neighbor-joining tree of HIV-1 *env* gp41 (n = 65) and protease (73) sequences collected in 1986. The Burkino Faso sequences are identified by the prefix, BF. Sequences from mixed HIV-1/HIV-2 infections are highlighted in gray. The reference HIV-1 group M and CRF sequences, respectively, have prefixes A, B, C, D, F1, F2, G, H, J, K, and potential L to denote the subtype or sub-subtype, or number indicating which CRF they represent, i.e., 1 for CRF01, 2 for CRF02, etc. The position of the outgroup (SIV-cpz sequence) is not shown. The numbers on nodal branches represent the bootstrap values (out of 100 replicates); only values 68% or greater are shown. The scale bar indicates an evolutionary distance of 0.1 nucleotides per site. Vertical distances are for clarity only.

Phylogenetic analysis of the 65 HIV-1 gp41 sequences from monotypic and heterotypic infections demonstrated that 37 (56.9%) were subtype G in that region, but further stongly subclustered with CRF06_cpx reference sequences; likewise, 25 (38.5%) clustered with subtype A sequences yet further subclustered with CRF02_AG reference strains (Figure 1A). Two (3.1%) were also subtype A but further subclustered with CRF09_cpx reference strains, and 1 (1.5%) clustered with subtype A reference sequences without any further subclustering. Similarly, phylogenetic analysis of the PR gene region of 73 sequences revealed 52 (71.2%) strongly subclustered with CRF06_cpx reference sequences, 15 (20.5%) subclustered with CRF02_AG, 5 (6.8%) closely related sequences clustered with subtype A sequences and 1 (1.4%) was a unique strain that clustered within the B/D lineage but basal to the node connecting the two (Figure 1B). Parallel analysis of HIV-1 PR and *env* gp41 gene regions, for the 14 strains with sequences available from both genes, revealed CRF concordance in 12 (85.7%) viruses, with 3 CRF02_AG(PR)/CRF02_AG(gp41) and 9 CRF06_cpx(PR)/CRF06_cpx(gp41). Of those with discordance between the two gene regions, 2 had mosaic patterns of CRF06_cpx(PR)/CRF02_AG(gp41) (Table 1), demonstrating further recombination occurring between the two prevalent recombinant viruses. The distribution of HIV-1 subtypes, recombinants, and dual infections is shown in Table 1. One limitation of our study is that we were only able to amplify fairly short gene regions due to small serum volumes and degradation to the HIV RNA since collection in the mid-1980s. While we tried to amplify both HIV-1 gp41 and PR for HIV-1 serologically determined homotypic infections, HIV-2 PR and INT for HIV-2 homotypic infections, and both for HIV-1/HIV-2 heterotypic infections, for most samples we only had success with one of the two gene regions.

**Figure 2:**
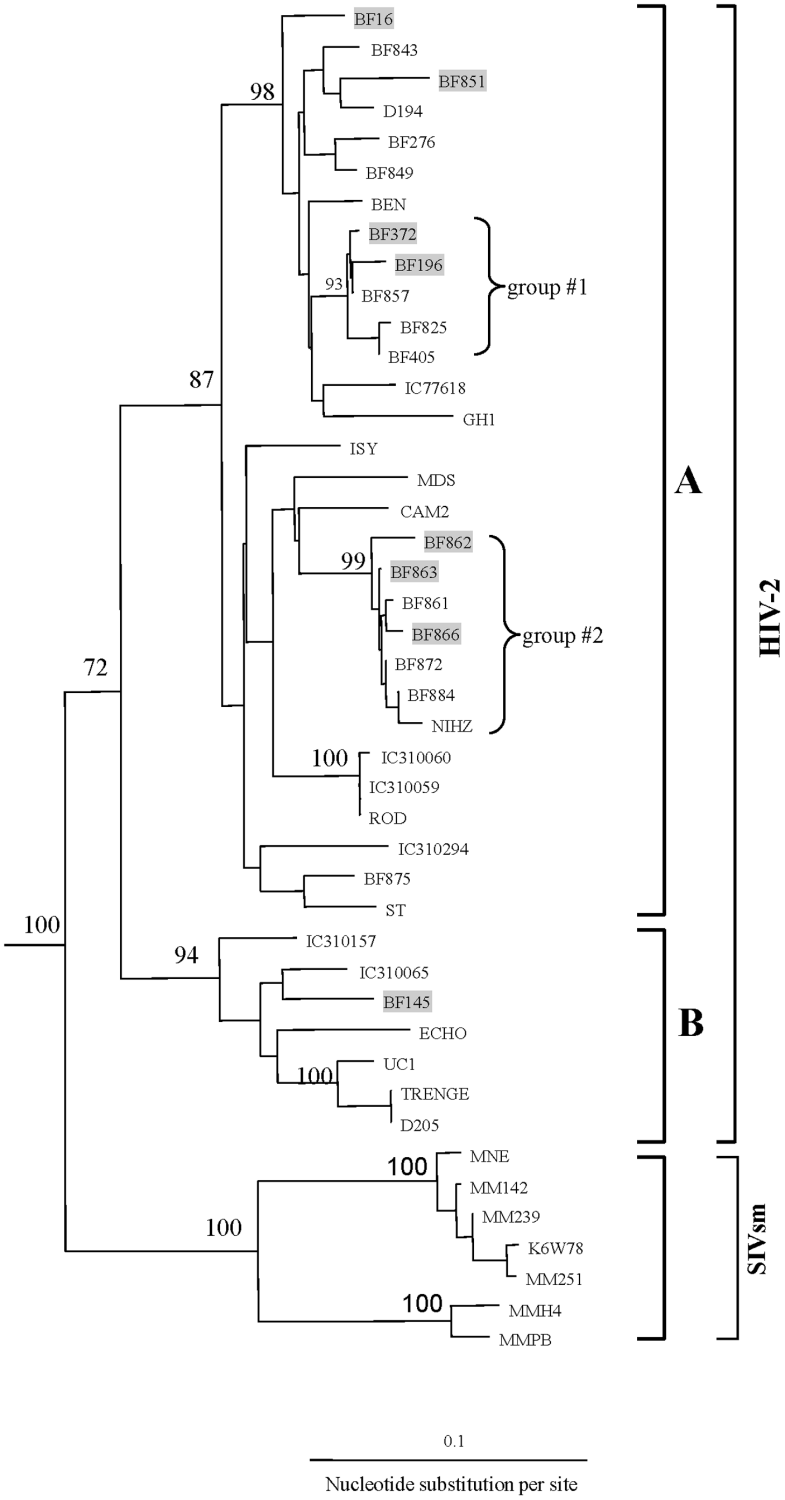
Neighbor-joining tree of 18 HIV-2protease sequences; the prefix BF represents sequences recovered from Burkina Faso patients in 1986. For comparison, 7 1980s sequences from Ivory Coast were included in the tree and contain the prefixes IC. Highlighted sequences in gray were from mixed HIV-1/HIV-2 infections; the remainders were from monotypic HIV-2 infections; groups #1 and #2 within subtype A represent distinct subclusters of closely related sequences. Viruses from sooty mangabeys are shown (SIVsm). The position of the outgroup (SIV-cpz sequence) is not shown. The number on the node indicates the bootstrap value; only values 68% or greater are shown. The scale bar indicates an evolutionary distance of 0.1 nucleotides per site. Vertical distances are for clarity only.

Phylogenetic analysis of 19 HIV-2 PR sequences from monotypic and heterotypic infections classified 17 sequences as group A (89.5%) and 2 as group B (10.5%) (Figure 2), with one of the group B being an INT sequence (Table 1). HIV-2 group A sequences were identified in all 10 HIV-2 monotypic infections. From the 8 HIV-1/HIV-2 heterotypic infections, HIV-1 strains were represented by 2 CRF06_cpx(PR) (25%), 1 CRF02_AG(PR) (12.5%), 1 CRF06_cpx(gp41) (12.5%), 3 CRF06_cpx(PR)/CRF06_cpx(gp41) (37.5%) and 1 subtype A in PR (12.5%) (Table 1). From those 8 heterotypic HIV-1/HIV-2 infections, HIV-2 group A was present in 7 (87.5%) and group B in 1 (12.5%).

### Genetic Distance Analysis

We found the mean nucleotide distance among all 52 Burkina Faso HIV-1 CRF06_cpx(PR) sequences was 1.1% (range 0.1–4.1%) (Table 2). Similarly, there was a limited nucleotide divergence with a mean of 1.8% (range 0.3–3.8%) for the 15 CRF02_AG(PR) sequences (Table 2). Also, low genetic distances were observed for the 37 CRF06_cpx(gp41) sequences with a mean of 2.0% (range 0.3–6.3%), 25 CRF02_AG(gp41) with a mean of 3.5% (range 0.8–7.7%), and 2 CRF09_cpx (mean 0.3%) (Table 2).

**Table 2:** Genetic distances within HIV subtypes and CRFs.

Gene Region	n	Subtype	Distance (%)	
			Mean	Range
HIV-1 PR	15	CRF02	1.8	0.3–3.8
	52	CRF06	1.1	0.1–4.1
HIV-1 gp41	25	CRF02	3.5	0.8–7.7
	37	CRF06	2	0.3–6.3
HIV-2 PR	17	A	6.2	0.3–12.8
HIV-2 PR group #1	5	A	0.8	0.3–2.3
HIV-2 PR group #2	6	A	1.1	0.3–3.0

CRF, circulating recombinant forms.

Genetic distance analysis of the 17 group A HIV-2 PR sequences revealed a broad diversity (mean 6.2%, 0.3–12.8%) (Table 2). Eleven of the 17 HIV-2 PR group A sequences phylogenetically clustered into 2 distinct subgroups supported by high bootstrap value of 93% and 99%, respectively (Figure 2). Pairwise nucleotide divergence was small among sequences within these 2 groups: mean 0.8%, range 0.3–2.3% for group #1 and mean 1.1%, range 0.3–3.0% for group #2 (Table 2). Pairwise analysis of the remaining 6 unrelated HIV-2 sequences showed a relatively high range (mean 6.1%, range 2.0–12.8%) of nucleotide diversity due to either a long existence of HIV-2 strains in Burkina Faso or the separate introduction of these variants into the country.

## Discussion

A unique set of historical serum samples collected in 1986 from Burkina Faso provided us the opportunity to determine the types and distribution of HIV that were present in the early years of the epidemic in that country. Based on two gene regions, PR and *env* gp41 of HIV-1 and PR or INT of HIV-2, our results showed that recombinant viruses were co-circulating early in the epidemic with the predominant strain being CRF06_cpx, a complex recombinant virus consisting of genomic regions from subtypes A, G, J, and K. CRF02_AG was the next major strain, followed by subtype A and CRF09_cpx, another complex recombinant virus, as minor strains. In addition, HIV-1 and HIV-2 heterotypic and HIV-2 monotypic infections were also present, with HIV-2 group A present in the majority of these infections.

Phylogenetic analysis of nucleotide sequences showed that the early 1986 HIV-1 epidemic in Burkina Faso was driven by recombinant viruses, with CRF06_cpx and CRF02_AG causing the majority of infections. Although these samples came from one hospital in Quagadougou, almost thirty years later the same CRFs are still predominant and co-circulating today and causing infections in Burkina Faso, with no significant change in proportion between CRF06_cpx and CRF02_AG [62], [63]; thus, strongly suggesting the same strains that initiated the early epidemic. Similarly, Kalish et al [24] found the distribution of HIV-1 strains in Kinshasa, Zaire from 1984 through 1986 were composed of CRF01_AE, unique recombinant viruses, unclassifiable strains, and all the group M subtypes but B. Other researchers found almost 20 years later, that the type and proportion of the Kinshasa subtypes and recombinant viruses were strikingly similar [26], [64].

Some of the earliest known samples of HIV infection (ZR59 and DRC60) were traced back to Kinshasa, Zaire [28], [29]. Although Central Africa appears to be the epicenter of the HIV-1 pandemic, CRF02_AG and CRF06 were not reported in Kinshasa until 2004. Following the first report of CRF06_cpx in a Burkina Faso specimen (BFP90) in 1996 [38], [41] and later in Mali in 1999 [39], CRF06_cpx viruses have since been documented in other West African countries, including Senegal, Mali, Ivory Coast, Niger and Nigeria [40], [65]. It is unclear where CRF06_cpx arose. Some have speculated that it originated in Burkina Faso because of the early predominance of CRF06_cpx in the country [41]. However, CRF06_cpx is a complex recombinant with portions of HIV-1 subtypes A, G, J, and K. Since subtypes J and K have not been reported in West Africa, especially so early in the epidemic, it does not seem likely that it could have arisen from multiple recombination events in Burkina Faso. A more plausible explanation is that it may have evolved in West-Central Africa, the epicenter of the HIV pandemic, and radiated to the west. Alternately, the first recombination events could have taken place in West-Central Africa, for instance between viruses from subtypes J and K, and additional recombination events took place when people with these infections traveled to the west where subtypes A and G were commonly reported. Since we only had PR or gp41 sequences, and both of these gene regions for CRF06-cpx are subtype G, another interesting possibility might be that these viruses could represent parental subtype G strains from which the CRF06 arose. However, as we state above, we do not believe that CRF06 originated in West Africa, since viruses of subtypes J and K were only found in Central Africa in the mid-1980s [27] and are still unreported in West Africa. Furthermore, recombination had to occur multiple times to generate the CRF06 virus. The very high genetic relatedness of all our early CRF06 viruses in Burkina Faso, and their close evolutionary relationship to CRF06 reference sequences, supports the conclusion that CRF06 was most likely introduced into Burkina Faso a couple years before our sampling in 1986. Finally, CRF06 and CRF02 are currently co-circulating in Burkina Faso in roughly the same proportions as we found in 1986 [62], [63].

CRF02-AG was first identified in West Africa between 1989 and 1991 [66], [67], and soon became the prominent strain throughout much of West and West Central African countries, including Nigeria, Cameroon, Senegal, and Cote d'Ivoire [65], [68]–[72]. While some investigators have suggested that recombinant viruses have the potential to be more pathogenic, more transmissible, or more biologically fit [73]–[78], it still remains to be proven that recombinant viruses have a selective advantage [79]. West Africa in the early 1980s was not yet experiencing an HIV-1 epidemic, so it was easy for newly introduced HIV-1 strains to become established within high-risk groups and then radiate out into the rest of the population. It is interesting, though, that the two strains that appear to have driven the 1986 HIV-1 epidemic in Burkina Faso were the recombinant viruses CRF06_cpx and CRF02_AG.

While the range of intra-subtype diversity was high in Kinshasa, Zaire in 1984-1986 [27], the intra-CRF diversity was low among 1986 HIV-1 PR and gp41gene sequences from Burkina Faso, suggesting that this was a much newer epidemic. In Zaire, during the same time period, the mean range of intra-CRF and intra-subtype diversity spanned 9.6% through 19.7% in the *env* C2V3C3 gene region, yet the *env* gp41 genetic distances for CRF06 and CRF02 showed only an average nucleotide difference of 3.5% and 2.0%, respectively. While the gp41 region is more conserved than the C2V3C3 region of the envelope, it does not explain the huge differences seen between the Kinshasa and Ouagadougou divergence among sequences from each city. The small amount of genetic differences among the viruses within CRF06_cpx and CRF02_AG suggest that they were recently introduced into Burkina Faso as founder viruses, not long before their sampling in 1986. Since the genetic divergence is larger for CRF06_cpx in both gene regions analyzed, it suggests that CRF02_AG may have entered Burkina Faso as a founder virus shortly after CRF06_cpx. A somewhat similar finding of low diversity among two founder viruses, CRF01_AE and subtype B’ was observed from specimens collected shortly after their introduction into Thailand [80]. Thai national serosurveys allowed researchers to conclude that there were two separate introductions of subtype B’ and CRF01_AE by parenteral and sexual risk factors, respectively, approximately one year apart. Unfortunately, we do not have any data about risk factors that might have helped us explain how these recombinant viruses entered Burkina Faso, but they do appear to be sequential introductions occurring within a couple years of each other.

Although there are at least 8 HIV-2 groups [15]–[17], only A and B have become endemic throughout West Africa, with group A being the dominant strain, except in Cote d’Ivoire, where HIV-2 group B is prevalent [81]–[83]. In our study of HIV strains in Burkina Faso, we found monotypic HIV-1 or HIV-2 infections as well as heterotypic HIV-1 and HIV-2 infections. Similar to what has been observed previously in West Africa, HIV-2 group A was predominant in Burkina Faso whether in HIV-2 monotypic or HIV-1/HIV-2 heterotypic infections.

We found the mean PR genetic distance among Burkina Faso HIV-2 group A viruses was 6.2%, which is considerably more divergent than HIV-1 PR for CRF02_AG and CRF06_cpx (1.8% and 1.0%, respectively). Since HIV-1 replicates faster and diverges more quickly than HIV-2, this greater divergence of HIV-2 viruses in Burkina Faso suggests that the introduction of HIV-2 could have predated the introduction of HIV-1, which is consistent with observations in other West African countries [84], [85]. However, another explanation for the higher diversity of HIV-2 group A could be the separate introduction of divergent strains into Burkina Faso, carried in from other West African countries where HIV-2 was already endemic.

Analysis of HIV-2 phylogenetic trees from Burkina Faso showed two strongly related sequence subclusters within group A. Each subcluster defines groups of closely related sequences with very short branch lengths and small nucleotide differences among them. These clusters represent two founder group A viruses that entered Burkina Faso and subsequently spread rapidly, most likely due to the high-risk groups they entered. These types of transmission networks contributed significantly to establishing HIV-2 infections in Burkina Faso and throughout West Africa. In addition, phylogenetic analysis demonstrated the sequences in group #2 formed a strong monophyletic cluster (99% bootstrap support) with the reference sequence NIHZ, which was originally isolated in 1986 from Guinea Bissau [86]. Such a relationship suggests that some of the HIV-2 strains found in Burkina Faso might have their origins in Guinea Bissau, a country in West Africa with one of the highest prevalences of HIV-2 infections [87] or that NIHZ-like strains originated in Burkina Faso and helped establish the HIV-2 epidemic in Guinea Bissau.

In summary, our results provide new and unique insights into the HIV epidemic in West Africa during this early period in the global pandemic. Based on sequences from only 138 HIV strains, it appears that CRF02_AG and CRF06_cpx were founder viruses that initiated the early HIV-1 epidemic in Burkina Faso. We also provide characterization of early HIV-2 sequences and document that while group A was predominant, both groups A and B variants were found in both HIV-2 monotypic and mixed HIV-1/HIV-2 infections. This information is critical for understanding the newly emerging HIV epidemic in Burkina Faso and for monitoring the dissemination and evolution of HIV-1 and HIV-2 strains in Burkina Faso, as well as throughout West Africa.
